# Identification of a Novel Prognostic Signature of Genome Instability-Related LncRNAs in Early Stage Lung Adenocarcinoma

**DOI:** 10.3389/fcell.2021.706454

**Published:** 2021-07-16

**Authors:** Bo Peng, Huawei Li, Ruisi Na, Tong Lu, Yongchao Li, Jiaying Zhao, Han Zhang, Linyou Zhang

**Affiliations:** ^1^Department of Thoracic Surgery, The Second Affiliated Hospital of Harbin Medical University, Harbin, China; ^2^Second Clinical College of Medicine, Harbin Medical University, Harbin, China

**Keywords:** long non-coding RNAs, genome instability, lung adenocarcinoma, prognosis, gene signature

## Abstract

**Background:**

Increasing evidence has demonstrated that long non-coding RNAs (lncRNAs) play a crucial part in maintaining genomic instability. We therefore identified genome instability-related lncRNAs and constructed a prediction signature for early stage lung adenocarcinoma (LUAD) as well in order for classification of high-risk group of patients and improvement of individualized therapies.

**Methods:**

Early stage LUAD RNA-seq and clinical data from The Cancer Genome Atlas (TCGA) were randomly divided into training set (*n* = 177) and testing set (*n* = 176). A total of 146 genomic instability-associated lncRNAs were identified based on somatic mutation profiles combining lncRNA expression profiles from TCGA by the “limma R” package. We performed Cox regression analysis to develop this predictive indicator. We validated the prognostic signature by an external independent LUAD cohort with microarray platform acquired from the Gene Expression Omnibus (GEO).

**Results:**

A genome instability-related six-lncRNA-based gene signature (GILncSig) was established to divide subjects into high-risk and low-risk groups with different outcomes at statistically significant levels. According to the multivariate Cox regression and stratification analysis, the GILncSig was an independent predictive factor. Furthermore, the six-lncRNA signature achieved AUC values of 0.745, 0.659, and 0.708 in the training set, testing set, and TCGA set, respectively. When compared with other prognostic lncRNA signatures, the GILncSig also exhibited better prediction performance.

**Conclusion:**

The prognostic lncRNA signature is a potent tool for risk stratification of early stage LUAD patients. Our study also provided new insights for identifying genome instability-related cancer biomarkers.

## Introduction

Non-small cell lung cancer (NSCLC) is a lethal cancer that causes over 1 million deaths a year (Torre et al., 2015; Yan et al., 2020; Zhu et al., 2021). Recently, the rising incidence of lung adenocarcinoma (LUAD), a major type of NSCLC, has become such a concerning issue that tends to surpass lung squamous carcinoma (Tong et al., 2018). The lack of early predictive biomarkers hurdles early screening and partly contributes to the stunning mortality. Although multidisciplinary approaches targeting cancer have achieved significant advancements, the overall prognosis for LUAD patients remains poor (Miller et al., 2019). As lung cancer screening is increasingly performed, more early stage LUAD patients are diagnosed. Therefore, exploring novel prognostic biomarkers should be paid much attention to in order to make risk stratification and to offer the optimal therapy for LUAD patients.

Genome instability, identified as mutations higher than normal rates, including tumor-specific DNA repair defects, DNA damage, and a failure to stop the cell cycle before the damaged DNA are transmitted to daughter cells (Lord and Ashworth, 2012; Tubbs and Nussenzweig, 2017), takes on the role of a double-edged sword in a number of biological processes; mutations facilitate evolution to a certain extent as a source of natural selection. Besides, genomic instability is such a prevalent characteristic of tumors that can be possibly deemed as an outcome predictor, and the accumulation of mutation is related to tumor progression and survival (Suzuki et al., 2003; Ottini et al., 2006). The underlying mechanisms of genomic instability have not been entirely elucidated yet. According to limited evidence, molecular signature has the potential for quantitative measurement of genomic instability (Tam et al., 2018). Wang et al. (2018) identified a genome instability-related 10-miRNA signature that is associated with prognosis of ovarian cancer (OV) (Zeng et al., 2018; Zhang et al., 2019). A subsequent study revealed mouse double minute 2 (MDM2) attenuated transcriptional inhibition of transcription factor HBP1 in the expression of its target genes via ubiquitination, which contributes to genome instability and, ultimately, tumorigenesis (Cao et al., 2019). During the past decade, genome-wide sequencing and microarray profiling have prompted the discovery of prognostic factors such as long non-coding RNAs (lncRNAs). LncRNAs refer to RNAs without protein-coding potential that have more than 200 nucleotides, involved in the survival, proliferation, migration, and genomic stability of cells (Moran et al., 2012; Iyer et al., 2015; Bao et al., 2019; Yao et al., 2019; Zhou et al., 2019). Recently, several lncRNA signatures have been established for predicting the prognosis of NSCLC patients, whereas the potential biological process and clinical significance of genome instability-associated lncRNAs in cancers remain unknown currently (Zheng et al., 2017; Lin et al., 2018; Li et al., 2020; Sun et al., 2020).

In this study, we searched two public databases, The Cancer Genome Atlas (TCGA) and the Gene Expression Omnibus (GEO), to construct a prognostic genome instability-associated lncRNA signature for early stage LUAD. A six-lncRNA signature with reliable prognostic performance was identified, which can be an indicator of genomic instability and improve patient stratification, thereby promoting personalized treatment decisions.

## Materials and Methods

### Data Collection and Study Design

Data of patients with early stage LUAD regarding the RNA-seq FPKM (fragments per kilobase of exon per million mapped fragments), clinical features, and somatic mutation were retrieved from TCGA database^1^. The patients who survived less than 30 days were removed from the cohorts. The key information of enrolled patients including paired lncRNA and mRNA expression profiles, somatic mutation, survival time, and clinicopathological features was extracted. A total of 353 enrolled patients were randomly allocated to two groups, training set (177 patients) and testing set (176 patients), separately. The clinical and pathological information is briefly presented in Table 1.

**TABLE 1:** Clinical characteristics of three early stage LUAD patient sets.

**Covariates**		**Training set (*n* = 177)**	**Testing set (*n* = 176)**	**TCGA set (*n* = 353)**	***p*-value**
Age, no (%)	≤65	87 (49.15)	79 (44.89)	166 (47.03)	0.621
	>65	86 (48.59)	89 (50.57)	175 (49.58)	
	unknow	4 (2.26)	8 (4.55)	12 (3.4)	
Gender, no (%)	female	104 (58.76)	90 (51.14)	194 (54.96)	0.183
	male	73 (41.24)	86 (48.86)	159 (45.04)	
T stage, no (%)	T1	64 (36.16)	58 (32.95)	122 (34.56)	0.602
	T2–3	113 (63.84)	118 (67.05)	231 (65.44)	
N stage, no (%)	N0	139 (78.53)	138 (78.41)	277 (78.47)	0.915
	N1	34 (19.21)	36 (20.45)	70 (19.83)	
	unknow	4 (2.26)	2 (1.14)	6 (1.7)	
Pathologic stage, no (%)	I	121 (68.36)	127 (72.16)	248 (70.25)	0.507
	II	56 (31.64)	49 (27.84)	105 (29.75)	

We searched the GEO database for another independent early stage NSCLC data set GSE50081^2^ (Der et al., 2014). After eliminating patients with other pathological types, we included the remaining 127 early stage LUAD patients with complete expression profiles and clinical data in the GSE50081 data set for further validation.

**FIGURE 1:**
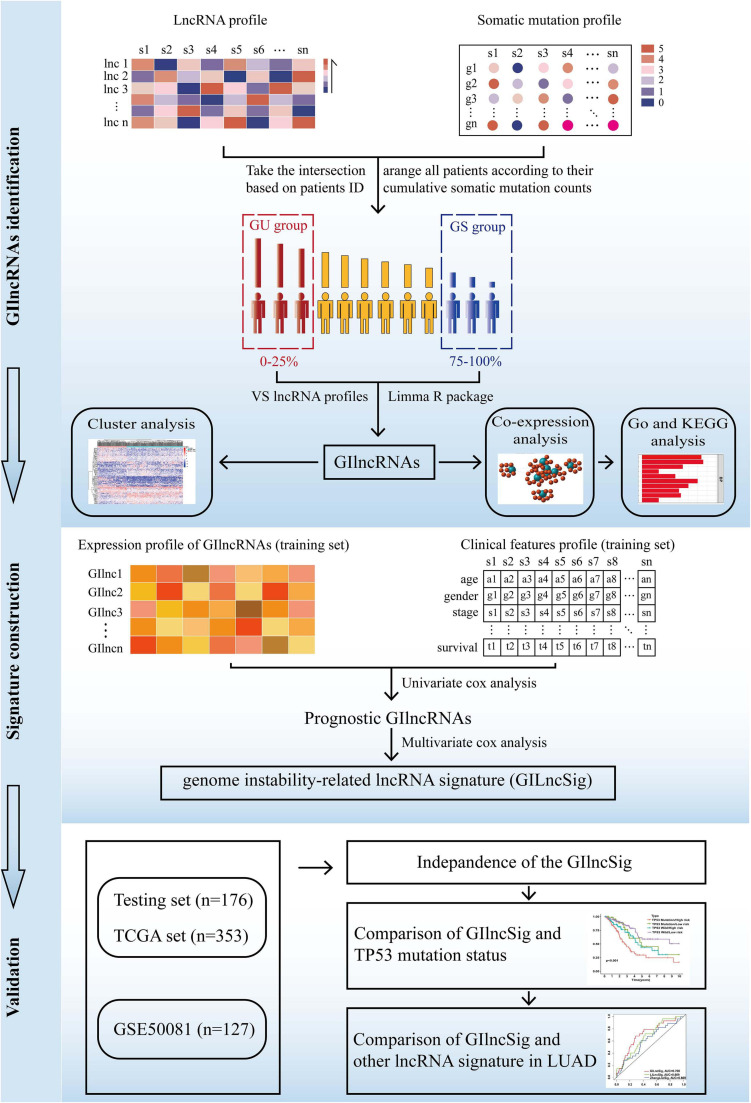
The flow diagram of this study.

The present study utilized differentially expressed lncRNA-based univariate Cox proportional regression analysis for prognostic lncRNAs in the training set (*p* value < 0.05). Then, a risk score model was used to establish the prognostic signature, and the testing set, the total TCGA set, and the GSE50081 data set were used to validate it. The study flowchart is illustrated in Figure 1.

### Genome Instability-Associated LncRNA Profiles

In order to study genome instability-associated lncRNAs, the somatic mutation information of 361 early stage LUAD patients and paired lncRNA expression profiles were obtained from downloaded TCGA data. We firstly calculated the total counts of somatic mutations of each patient. Secondly, we arranged subjects in descending order by counts of somatic mutations. The genomic unstable (GU) group refers to the former 25% subjects. Similarly, the genomic stable (GS) group refers to the last 25%. We conducted Wilcoxon test analysis with “limma R” package to compare the expression of lncRNAs between the two groups above to find genome instability-associated lncRNAs (absolute values of log fold change (FC) > 1 and false discovery rate (FDR) adjusted *p* < 0.05).

### Cluster Analysis and Co-expression Analysis

According to expression quantification of genome instability-associated lncRNAs, all patients were therefore divided into the GU-like cluster and the GS-like cluster by “limma R” and “sparcl R” packages. Pearson’s correlation test was applied. The 10 mRNAs with the strongest correlation, computed by Pearson’s correlation, were identified as co-expressed lncRNA-related ones for further functional enrichment analysis.

### Development of Prognostic Signature

The association between overall survival and the expression of genome instability-related lncRNAs was examined by univariate and multivariate Cox proportional hazard regression. We further aimed to predict prognosis; thereby, a genome instability-related lncRNA signature (GILncSig) was constructed. We computed the risk score for every early stage LUAD subject based on the expression values of prognostic genome instability-associated lncRNAs and their relevant coefficient:

$$G\,I\,L\,n\,c\,S\,i\,g\,\left(r\,i\,s\,k\,s\,c\,o\,r\,e\right)=\sum_{i=1}^{n}c\,o\,e\,f\,\left(\text{lncRNAi}\right)*e\,x\,p\,r\,\left(\text{lncRNAi}\right)$$

where *n* represents the number of prognostic lncRNAs; coef (lncRNAi), the regression coefficient of lncRNAi in the multivariate Cox analysis; and expr (lncRNAi), the expression value of lncRNAi. The high-risk group with high GILncSig was distinguished from the low-risk group according to the median risk score.

#### Function Enrichment Analysis

We performed Gene Ontology (GO) terms and Kyoto Encyclopedia of Genes and Genomes (KEGG) pathway enrichment analyses based on co-expressed mRNAs to discover the potential biological functions and risk pathway of genome instability-associated lncRNAs. ‘‘clusterProfiler R,’’ ‘‘enrichplot R,’’ and ‘‘ggplot2 R’’ packages were used for the above analyses in the R program. We performed Gene Set Enrichment Analysis (GSEA) (version 4.0.3)^3^ to explore the potential biological processes and risk pathways between the low- and high-risk groups calculated from our prognostic signature. The significant biological processes and pathways were enriched with FDR < 0.05. The c2.cp.kegg.v7.4.symbols.gmt was chosen as the reference file.

### Statistical Analyses

R-version 4.0.3 was used for statistical analysis. We utilized the Kaplan–Meier plot to estimate differences in survival rate and median survival. To measure statistical significance, we used the log-rank test. Moreover, we wondered whether the GILncSig was independent of other key clinicopathological features; therefore, multivariate Cox regression analysis and stratification analysis were performed. To examine the GILncSig performance, we compared the areas under the receiver operating characteristic (ROC) curves (AUC).

We used the “survival R” and “survminer R” packages in R software to perform survival analyses. The development of prediction signature was performed by “survival R” package, “survminer R” package, “caret R” package, “glmnet R” package, “pheatmap R” package, “time ROC R” package, “ggplot2 R” package, and the “ggpubr R” package.

## Results

### Identifying Genomic Instability-Related LncRNAs in Early Stage LUAD Subjects

**FIGURE 2:**
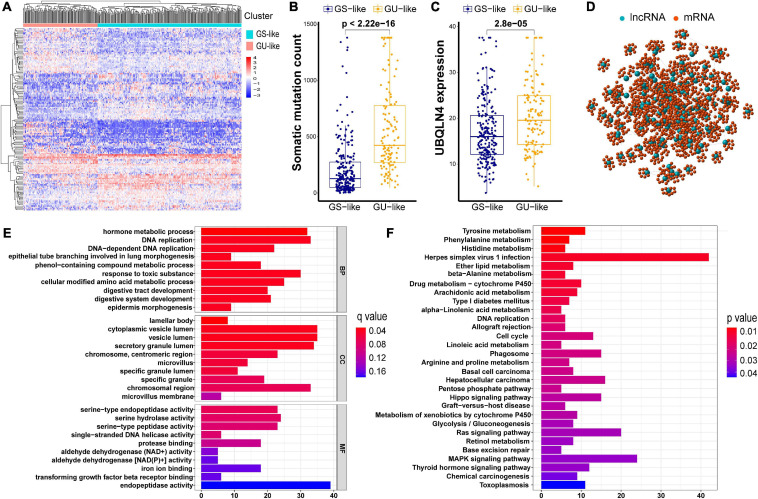
Cluster analysis of all patients in TCGA set and functional annotations of 146 lncRNAs. **(A)** Unsupervised clustering of all patients in TCGA set according to the expression quantification of 146 lncRNAs. The GU-like group is described in the left red cluster, meanwhile the GS-like group is described in the right blue cluster. **(B)** Comparison of somatic cumulative mutation counts between the GS-like group and the GU-like group. **(C)** Comparison of UBQLN4 between the GS-like group and the GU-like group. **(D)** Co-expression network of genomic instability-related lncRNAs and mRNAs. **(E)** GO analysis for lncRNA co-expressed mRNAs. **(F)** KEGG analysis for lncRNA co-expressed mRNAs.

As described in Methods, we distributed the former 25% (*n* = 61) and the last 25% (*n* = 61) of the early stage LUAD subjects to the GU and GS groups, respectively, with regard to the total counts of somatic mutations. A total of 146 differentially expressed lncRNAs with their absolute values of log FC > 1 and FDR adjusted *p* < 0.05 were identified by comparing the mean values of each lncRNA expression. Among all the lncRNAs studied, in the GU group, there were 73 upregulated and 73 downregulated (Supplementary Table 1). A heatmap of 20 upregulated lncRNAs and 20 downregulated lncRNAs with the most significant differences is shown in Supplementary Figure 1. Furthermore, we clustered subjects from TCGA set into the GU-like (higher cumulative somatic mutations) and GS-like (lower cumulative somatic mutations) groups. These two groups were distinguished by the expression of a total of 146 lncRNAs. As shown in Figure 2A, there are significantly different somatic mutation patterns between the two groups. We found that in the GU-like group, the degree of cumulative somatic mutation and UBQLN4, a driver gene of genomic instability, were much higher than those of the GS-like group (*p* < 0.001, Figures 2B,C).

In order to investigate the biological functions and potential risk pathway of the 146 lncRNAs, we performed GO and KEGG pathway enrichment analyses. We first identified 10 mRNAs with the strongest correlation for each of the above 146 lncRNAs, As shown in the lncRNA–mRNA co-expression network, the linked lncRNA and mRNA showed that there were correlations between them (Figure 2D). According to the GO analysis, the biological processes were mainly involved in the development and maintenance of genomic instability, including DNA replication and DNA-dependent DNA replication (Figure 2E). Moreover, most enriched pathways in the KEGG analysis were linked to amino acid metabolism (Figure 2F). Accumulatively, there were a total of 146 lncRNA candidates as genome instability-associated lncRNAs.

### Development of GILncSig in the Training Set

**FIGURE 3:**
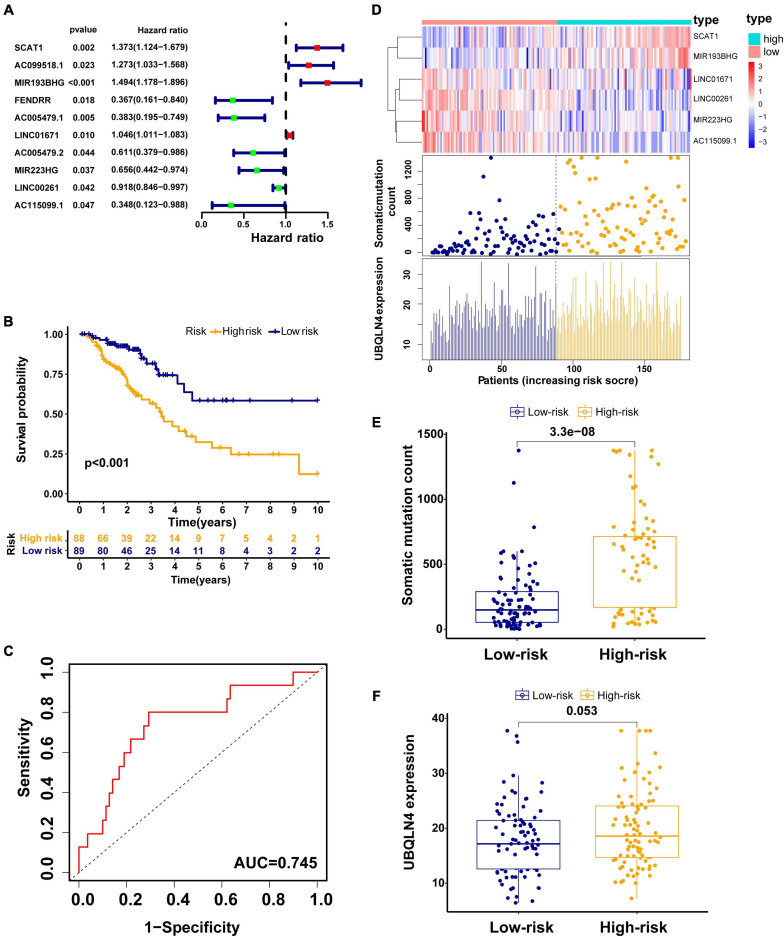
Construction of the GILncSig for predicting prognosis in the training set. **(A)** The forest plot of 10 genomic instability-related lncRNAs related to survival. **(B)** Kaplan–Meier curve in different risk groups stratified by GILncSig in the training set. **(C)** Time–ROC curve analysis of the GILncSig in the training set at 3 years. **(D)** Change trend of GILncSig, somatic mutation, and UBQLN4 with an increasing order of risk score. Comparison of the total somatic mutation counts **(E)** and UBQLN4 expression **(F)** between the two risk groups stratified by GILncSig in the training set. Horizontal lines: median values.

To explore the prognostic value of 146 candidate genome instability-associated lncRNAs, 353 patients in the TCGA set were randomly allocated to two groups, training set (177 patients) and testing set (176 patients), separately. Subsequent statistical analysis indicated that there were no significant differences in clinicopathological covariates between the training set and the testing set (Table 1). According to our findings, there were 10 genome instability-related lncRNAs significantly related to overall survival based on results from univariate Cox regression analysis, of which the forest plot was constructed (Figure 3A). We next performed multivariate Cox proportional hazards regression analysis and identified 6 of 10 candidate lncRNAs as independent prognostic lncRNAs (Table 2). Finally, based on GILncSig, the six genome instability-related lncRNAs won the nomination as prognosis predictor for early stage LUAD. A risk-score formula was constructed as follows: risk score = 0.229 × expression quantity of SCAT1 + 0.225 × expression quantity of MIR193BHG + 0.069 × expression quantity of LINC01671 + (−0.367) × expression quantity of MIR223HG + (−0.099) × expression quantity of LINC00261 + (−0.673) × expression quantity of AC115099.1. Three lncRNAs with positive coefficients tended to be risky factors, while three lncRNAs with negative coefficients tended to be protective factors. We then calculated the risk score for each patient in the training set and classified them into the high-risk group (*n* = 88) and the low-risk group (*n* = 89) using the median risk score (1.250) as a cutoff value. We discovered that the low-risk group surpassed the high-risk group in overall survival rates according to the Kaplan–Meier analysis (*p* < 0.001, Figure 3B). The GILncSig reached an area under curve (AUC) of 0.745 in terms of time-dependent ROC curves analysis (Figure 3C). We next ranked the patients in increasing order according to their risk scores and observed changes in trend of GILncSig, numbers of somatic mutation, and expression values of UBQLN4 (Figure 3D). The patients with high-risk scores showed a tendency for increased counts of somatic mutation and expression level of UBQLN4. Further comparison analysis indicated the high-risk group had more somatic mutations than the low-risk group (*p* < 0.001, Figure 3E). Similarly, the high-risk group appeared to exhibit higher UBQLN4 level, although this did not achieve statistical significance (*p* = 0.053, Figure 3F).

**TABLE 2:** Multivariate Cox regression analysis of six prognostic lncRNAs.

**LncRNAs**	**Coefficient**	**HR**	**HR (95%CI)**	***p*-value**
SCAT1	0.229	1.257	1.000–1.580	0.050
MIR193BHG	0.225	1.252	0.951–1.647	0.109
LINC01671	0.069	1.072	1.039–1.106	<0.001
MIR223HG	−0.367	0.693	0.465–1.031	0.071
LINC00261	−0.099	0.906	0.830–0.988	0.026
AC115099.1	−0.673	0.510	0.199–1.308	0.161

*HR, hazard ratio; CI, confidence interval.*

### Validation of the GILncSig in Testing Set, TCGA Set, and Another Independent Data Set With Microarray Platform

**FIGURE 4:**
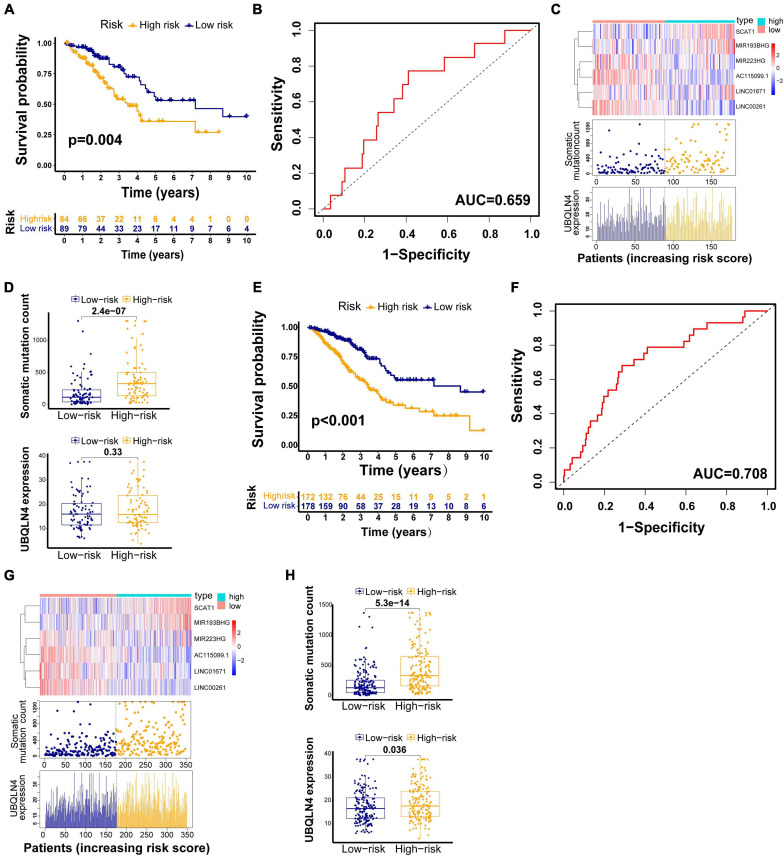
GILncSig evaluation on the validation sets. Kaplan–Meier curve of survival in the testing set **(A)** and TCGA set **(E)**. Time–ROC curves at 3 years in the testing set **(B)** and TCGA set **(F)**. Change trend of GILncSig, somatic mutation, and UBQLN4 in the testing set **(C)** and TCGA set **(G)**. Comparison of the total somatic mutation counts and UBQLN4 level between the two risk groups stratified by GILncSig in the testing set **(D)** and TCGA set **(H)**.

The testing set and TCGA set were firstly used to validated our findings obtained from the training set. Among 176 patients in the testing set, classified according to a cutoff value (1.250), the high-risk group (*n* = 85) had much poorer survival rate than low-risk group (*n* = 91) (*p* = 0.004, Figure 4A). The AUC of the testing set drawn from the time-dependent ROC curve analysis was 0.659 (Figure 4B). The testing set showed similar patterns of the GILncSig expression, somatic mutations, and UBQLN4 expression to the training set (Figure 4C). Moreover, distinct difference in the somatic mutation counts was found between the high-risk group and the low-risk group (*p* < 0.001, Figure 4D). The high-risk patients tended to express higher UBQLN4 compared with patients in low-risk group. Significance was not reached but approached (*p* = 0.33, Figure 4D).

We also observed similar results in the TCGA set. Patients in the TCGA set were partitioned into two risk groups, high-risk group (*n* = 173) and low-risk group (*n* = 180), according to a cutoff risk score (1.250) obtained from the training set. The high-risk group had much lower overall survival rates than the low-risk group (*p* < 0.001, Figure 4E). The AUC of the TCGA set drawn from the time-dependent ROC curves analysis was 0.708 (Figure 4F). The distribution patterns of the GILncSig expression, somatic mutation count, and expression of UBQLN4 were consistent with the above results (Figure 4G). We observed much more somatic mutation in the high-risk group (*p* < 0.001, Figure 4H). In addition, we found significant differences in the UBQLN4 expression between the two risk groups (*p* < 0.001, Figure 4H).

**FIGURE 5:**
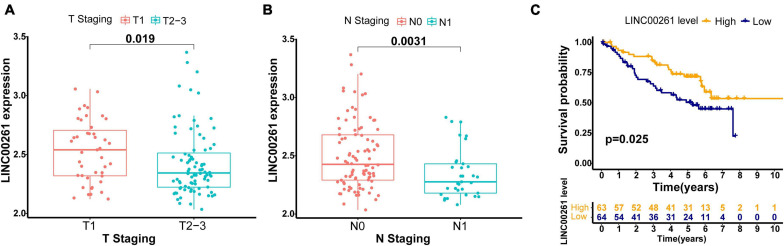
Performance assessment of the GILncSig in independent external GEO data set (GSE50081). **(A)** Comparison of LINC00261 expression levels between the two groups with different T stage in the GSE50081 set. **(B)** Comparison of LINC00261 expression levels between the two groups with different lymph node status in the GSE50081 set. **(C)** Kaplan–Meier curve for the LINC00261 expression in the GSE50081 set.

To further validate the robustness of the GILncSig, we searched for other independent data sets with microarray platform from the GEO database. After data reannotation, we finally found that only one lncRNA (LINC00261, a protective factor) of the GILncSig was covered by GSE50081 with lncRNA expression profiles and paired clinicopathological information of 127 early stage LUAD patients. Therefore, we studied the relationship of LINC00261 and outcome of early stage LUAD patients in GSE50081. LINC00261 expression and tumor T stage were strongly related (*p* = 0.019, Figure 5A). Furthermore, patients without lymph node metastasis had an increased tendency to have higher LINC00261 than those with lymph node metastasis (*p* = 0.003, Figure 5B). As could be expected, higher LINC00261 expression was significantly associated with better prognosis (*p* = 0.025, Figure 5C). In addition, no correlation between LINC00261 and age or gender was observed (Supplementary Figure 2). As expected, the results above agreed with findings from both the training and testing sets.

### Independence of the GILncSig From Other Clinicopathological Factors

We conducted multivariate Cox regression analyses on age, gender, pathologic stage, and prognostic risk score of GILncSig to investigate the independent prognostic effect of GILncSig. After adjusting for age, gender, and pathologic stage, we observed that GILncSig was an independent factor for overall survival of early stage LUAD in each set (Table 3). Moreover, pathologic stage was found to be significant in all three sets according to the multivariate Cox regression analysis, indicating that it could be another independent prognostic factor.

**TABLE 3:** Univariate and Multivariate Cox regression analysis of the GILncSig and prognosis.

**Variables**		**Univariate model**			**Multivariate model**		
		**HR**	**95% CI**	***p*-value**	**HR**	**95% CI**	***p*-value**
**Training set (*n* = 177)**							
Age		1.006	0.977–1.035	0.706			
Gender	Male/Female	1.326	0.766–2.296	0.313			
Stage	II/I	2.398	1.378–4.173	0.002	2.236	1.271–3.934	0.005
Risk score	High/Low	1.050	1.028–1.073	<0.001	1.044	1.022–1.068	<0.001
**Testing set (*n* = 176)**							
Age		1.038	1.008–1.070	0.014	1.041	1.009–1.074	0.011
Gender	Male/Female	1.064	0.634–1.784	0.815			
Stage	II/I	2.947	1.729–5.025	<0.001	3.086	1.803–5.284	<0.001
Risk score	High/Low	1.047	1.002–1.094	0.039	1.063	1.017–1.111	0.007
**TCGA set (*n* = 353)**							
Age		1.021	1.000–1.042	0.046	1.025	1.004–1.047	0.022
Gender	Male/Female	1.182	0.813–1.721	0.381			
Stage	II/I	2.625	1.794–3.843	<0.001	2.649	1.802–3.894	<0.001
Risk score	High/Low	1.043	1.025–1.060	<0.001	1.036	1.019–1.054	<0.001

*HR, hazard ratio; CI, confidence interval.*

**FIGURE 6:**
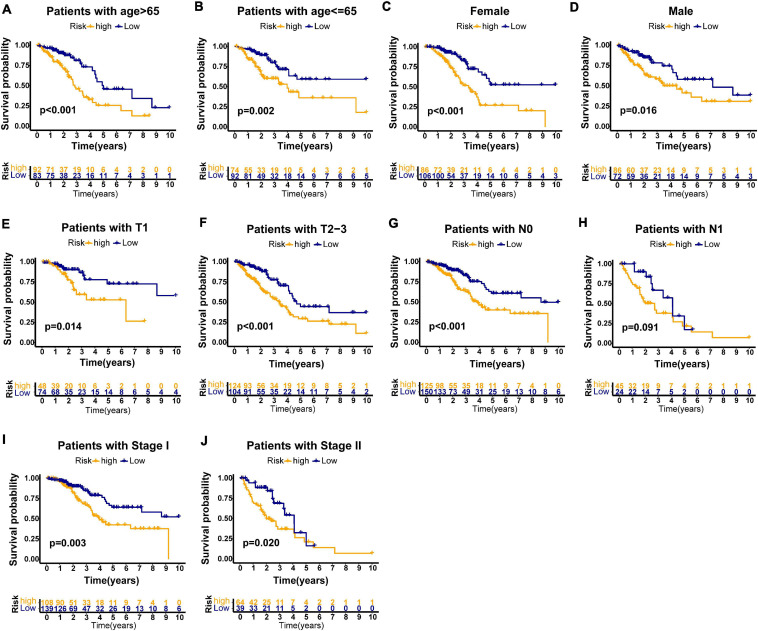
Stratification analyses by age, gender, T stage, N stage, and pathologic stage in all patients of TCGA set. Kaplan–Meier survival curve in different risk groups stratified by GILncSig for older patients **(A)** and young patients **(B)**; female patients **(C)**, and male patients **(D)**; patients with T1 stage **(E)** and patients with T2–3 stage **(F)**; patients with no lymph node metastasis **(G)** and patients with lymph node metastasis **(H)**; and patients with stage I **(I)** and patients with stage II **(J)**.

Furthermore, we performed stratification analysis to estimate whether our GILncSig is applicable to early stage LUAD patients with different clinical parameters. In the TCGA set, subjects got into groups of two according to a cutoff value (age = 65), a younger patient group (*n* = 166) and an older patient group (*n* = 175). Each group was further stratified into two risk groups according to calculated risk score by GILncSig. The overall survival rates between the high-risk group and the low-risk group in the older patient group (*p* < 0.001, Figure 6A) and the younger patient group (*p* = 0.002, Figure 6B) were significantly different. Furthermore, all subjects in the TCGA set were also classified into female (*n* = 192) and male groups (*n* = 158) based on gender. The GILncSig classified the female group and male group into the high-risk or low-risk group, respectively. Figures 6C,D indicated that the overall survival rates between the two risk groups were significantly different in the female group as well as in the male group (*p* < 0.001; *p* = 0.016). Using the same methodology, all patients were stratified with T stage, N stage, and pathologic stage followed by GILncSig classification, respectively. Among the six subgroups, differences of overall survival rates were observed between the high-risk and low-risk groups (*p* = 0.014, Figure 6E; *p* < 0.001, Figure 6F; *p* < 0.001, Figure 6G; *p* = 0.091, Figure 6H; *p* = 0.003, Figure 6I; *p* = 0.020, Figure 6J). Taken together, our findings suggested that the GILncSig could independently predict overall survival of early stage LUAD patients.

### Comparison of Prediction Performance Between the GILncSig and TP53 Mutation Status

**FIGURE 7:**
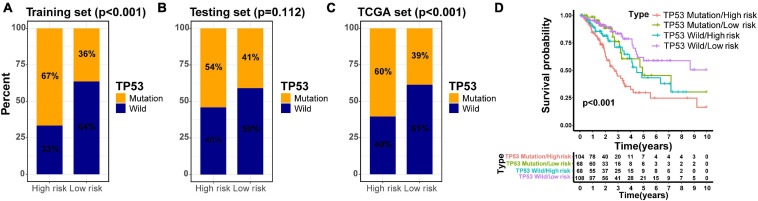
Joint survival analysis of the GILncSig and TP53 mutation status. TP53-mutator proportion in different risk groups stratified by GILncSig in three sets **(A–C)**. **(D)** Kaplan–Meier survival curve in four groups classified by the GILncSig and TP53 mutation status.

Previous investigations have demonstrated that tumor protein 53 (TP53) mutation has a negative prognostic role in the NSCLC patients (Lee et al., 2015; Deben et al., 2016; Zhao et al., 2019; Qin et al., 2020). To explore the distribution of TP53 mutation status in different risk groups divided by the GIlncSig, we identified the mutation status of TP53 in all TCGA patients based on somatic mutation profiles. Further study demonstrated that the high-risk group in the training set had more TP53 mutations than the low-risk group (*p* < 0.001, Figure 7A). Similarly, the results in the testing set and TCGA set confirmed the above findings, although the *p* value in the testing set was not considered significant enough (*p* = 0.112, Figure 7B; *p* < 0.001, Figure 7C). To compare the prediction performance between TP53 mutation status and the GILncSig, we classified the patients of TCGA set into four groups based on their TP53 mutation status and risk score, including TP53 mutation/high-risk group, TP53 mutation/low-risk group, TP53 wild/high-risk group, and TP53 wild/low-risk group. The survival curves of four groups indicated that the TP53 wild/low-risk group had much better overall survival than the TP53 mutation/high-risk group, while the overall survival rates were similar between the TP53 mutation/low-risk group and the TP53 wild/high-risk group (*p* < 0.001, Figure 7D). Therefore, our findings suggested that GILncSig might have almost equivalent prediction performance for overall survival compared with TP53 mutation status of patients in early stage LUAD.

### The GILncSig Predicts Outcome Better Than Existing LncRNA-Related Signatures

**FIGURE 8:**
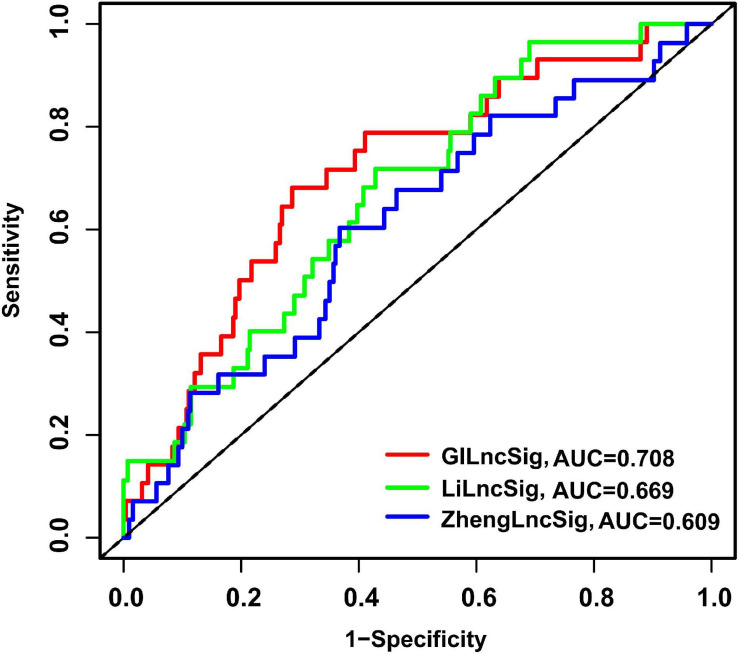
The ROC analysis of overall survival at 3 years for the GILncSig, ZhenglncSig, and LilncSig.

To assess the advantage of the GILncSig, we further made a comparison in the prediction activity of the GILncSig between the eight-lncRNA signature and seven-lncRNA signature derived from the study by Zheng (denoted hereafter as ZhenglncSig) (Zheng et al., 2017) and Li (denoted hereafter as LilncSig) (Li et al., 2020), respectively, in an assistance of the same TCGA set. Interestingly, for GILncSig, the AUC of overall survival at 3 years (AUC = 0.708) was higher than that of ZhenglncSig (AUC = 0.609) and LilncSig (AUC = 0.669) (Figure 8). Additionally, a smaller number of prognostic lncRNAs in the GILncSig was seen in our study than that in the ZhenglncSig and LilncSig. Because of this, the GILncSig seems to perform better in the role of predicting outcomes than other two recently published lncRNA signatures.

### GSEA of the High-Risk Group in Early Stage LUAD Patients Based on the GILncSig

In order to further elucidate the potential underlying pathways involved in the high- and low-risk groups calculated by the GILncSig in TCGA set, GSEA enrichment analysis was performed. It is noteworthy that 28 significant pathways were enriched in the high-risk group according to the cutoff: FDR < 0.05 (Supplementary Table 2). Of these, the cell cycle signaling pathway, nucleotide excision repair, homologous recombination, DNA replication, spliceosome, mismatch repair, and oocyte meiosis were enriched in the high-risk group (Supplementary Figure 3). However, there was no significantly enriched pathway in the low-risk group based on the cutoff value.

## Discussion

Because of unknown pathogenesis, LUAD is such a devastating disease that is not only the commonest pathological subtype but also with a frustrating 5-year overall survival rate at almost 20% (Wang et al., 2018; Miller et al., 2019). Therefore, individualized therapy intensification for high-risk patients in early stage LUAD is needed. In the past decade, genomic and transcriptional studies have pushed forward the work of cancer investigation to a great extent. In this study, a brand new six-lncRNA prognostic signature from early stage LUAD patients was confirmed by using the TCGA data set, an efficient approach to discover novel prognostic biomarkers and therapeutic target. The developed six-gene signature exhibits good performance to distinguish high-risk early stage LUAD patients for poorer prognosis, which may help with clinical treatment for patients with early stage LUAD.

As essential members of non-coding RNAs, the roles of lncRNAs in cellular homeostasis and cell proliferation, migration, and genomic stability have been illustrated in many previous studies (Huarte, 2015). Previous studies have identified several lncRNAs correlated with the outcome of NSCLC patients. For example, Li et al. (2020) established a predictive model for outcome based on seven immune-related lncRNAs. Moreover, Lin et al. (2018) identified a total of seven lncRNAs related to overall survival in NSCLC. In addition, the value of lncRNAs in evaluating the immune infiltrate of the tumor was illustrated, and an lncRNA signature (TILSig) associated with tumor immune infiltration to predict outcome of NSCLC was identified in the study by Sun et al. (2020). However, there is still little known about the genome instability-associated lncRNAs. A recent study has revealed two genome instability-associated lncRNAs (LINC02207 and RP11-358L4.1) with prognostic value in breast cancer patients, and the two lncRNAs are significantly correlated with somatic mutation phenotype. To further explore the potential effects of genome instability in tumors, we identified 146 genome instability-related lncRNAs. It is known that genome instability is caused by errors at different processes of the DNA cycle from replication to segregation, and DNA replication is tightly regulated at every stage from initiation to termination (Boos et al., 2012). We conducted functional analysis of mRNAs that co-expressed with the 146 lncRNAs and found that the genes were basically enriched in DNA replication and DNA-dependent DNA replication, involved in the maintenance of genomic instability.

As for the characteristics of the six lncRNAs correlated with genome instability based on our predictive signature, three-lncRNA (SCAT1, MIR193BHG, and LINC01671) expression was found to be a risky factor, while other three lncRNAs (MIR223HG, LINC00261, and AC115099.1) tended to be protective factors. This is the first time that almost every identified genome instability-related lncRNAs, except LINC00261, is demonstrated in cancer. Previous studies suggested that LINC00261 negatively regulates cellular proliferation of LUAD by activating the DNA damage response function as a tumor suppressor (Shahabi et al., 2019). In addition, downregulated LINC00261 was confirmed to be related to poor prognosis of gastric cancer. Further mechanism experiments *in vitro* indicated that LINC00261 suppresses GC metastasis by regulating epithelial–mesenchymal transition (Fan et al., 2016). This evidence effectively establishes the accuracy of our GILncSig. However, little knowledge about the other five lncRNAs can be acquired from recent studies. Well-designed studies should be performed to unlock their potential functions and mechanisms in cancers, which is helpful to unmask their role as therapeutic targets.

The widely accepted system to predict survival and assist treatment decision for LUAD patients is still the TNM staging system (Groome et al., 2007). However, it is not precise enough to predict individual survival and guide patient management. In our study, the GILncSig held great promise to predict the prognosis of LUAD patients. Further stratification analysis indicated that the GILncSig could stratify patients with same stage (T stage, N stage, and pathologic stage) into high- and low-risk groups and screen the patients with poor outcome. Of note, the result of the time-dependent ROC curve analysis indicated that the AUC values of the GILncSig were more than 0.60 in the training and testing sets, suggesting the accuracy of our research. Compared with other published lncRNA prognostic signatures of LUAD in terms of the ROC analysis, our signature seems to perform more accurately at predicting survival outcome as well. Thus, the GILncSig could be considered as a certain value to improve the prognosis and thus could provide better guidance of individualized treatment for patients with early stage LUAD.

Although the six-lncRNA signature is promising, limitations should be noted in this initial work. Firstly, the patient cohort of TCGA was from multiple institutions, and due to the limited information of somatic mutations, our sample size was not very large. The findings from our research need to be further verified in other data sets. Secondly, biological experiments, both *in vitro* and *in vivo*, on these predictive lncRNAs are required.

In conclusion, we constructed a risk-score signature containing six lncRNAs to predict the outcome of patients with early stage LUAD. Further analysis indicated that the GILncSig could be a prognostic indicator independent of other conventional clinicopathological variables. Further efforts, especially studies on the large, well-performed cohorts, are necessarily needed to improve the six-lncRNA signature.

## Data Availability Statement

The original contributions presented in the study are included in the article/Supplementary Material, further inquiries can be directed to the corresponding author/s.

## Author Contributions

BP conceived the research, downloaded and analyzed the data, and wrote the manuscript. HL participated in processing the data and guided the study. RN, TL, YL, JZ, and HZ participated in preparing the figures and revising the manuscript. LZ made manuscript revisions and approved the final draft. All authors contributed to the article and approved the submitted version.

## Conflict of Interest

The authors declare that the research was conducted in the absence of any commercial or financial relationships that could be construed as a potential conflict of interest.

## Footnotes

1 https://portal.gdc.cancer.gov/

2 https://www.ncbi.nlm.nih.gov/geo/query/acc.cgi?acc=GSE50081

3 http://www.broadinstitute.org/gsea
