# Supplementary material for: Identification of a Novel Prognostic Signature of Genome Instability-Related LncRNAs in Early Stage Lung Adenocarcinoma
Source: Front Cell Dev Biol. 2021 Jul 16;9:706454. doi: 10.3389/fcell.2021.706454 (PMC8324209; doi:10.3389/fcell.2021.706454)
Supplement: Supplementary file 1 [file Data_Sheet_1.PDF]

Supplementary Material

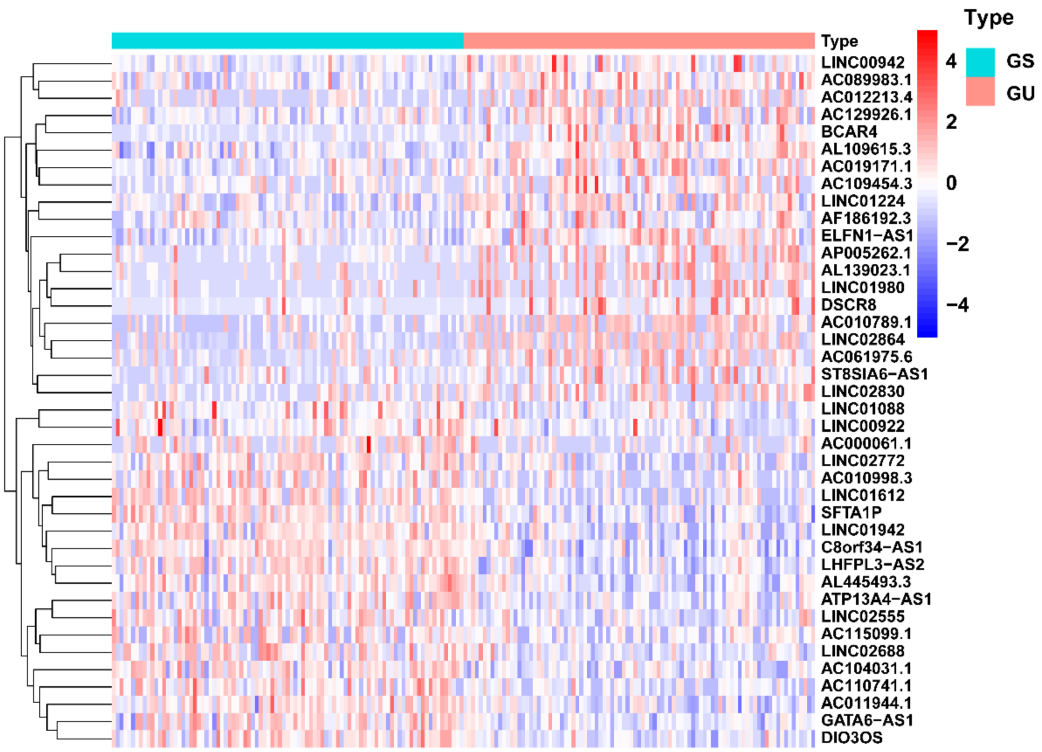

Supplementary Figure S1. A heatmap of 40 lncRNAs with the most significant differences between the GS group and GU group.

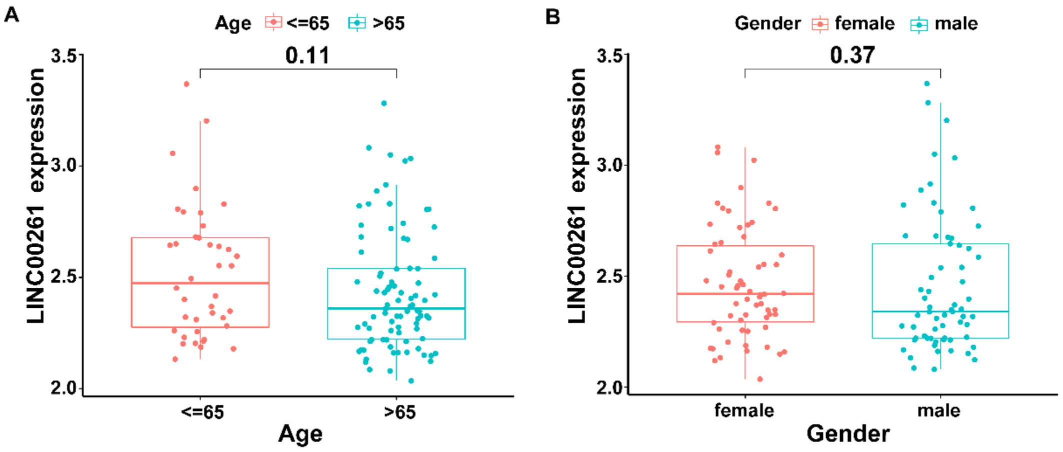

Supplementary Figure S2. Performance assessment of the GILncSig in independent external GEO data set (GSE50081). **(A)** Comparison of LINC00261 expression levels between the two groups with different age in GSE50081 set. **(B)** Comparison of LINC00261 expression levels between the two groups with different gender in GSE50081 set.

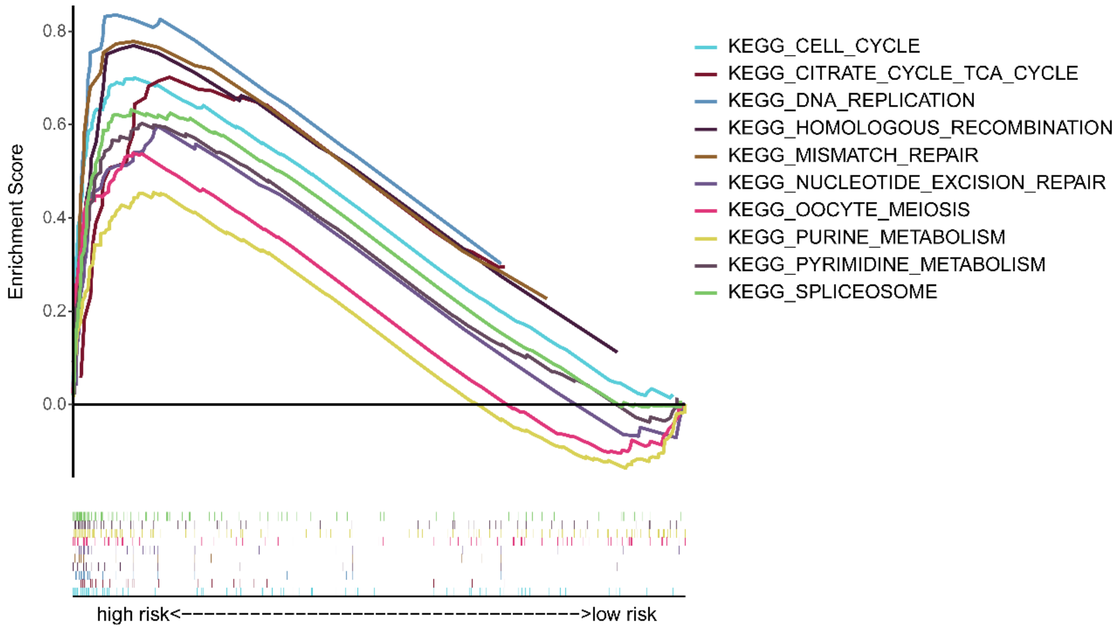

Supplementary Figure S3. Gene Set Enrichment Analysis (GSEA) of the high-risk group in early-stage LUAD patients based on the prognostic signature.

Supplementary Table S1. 146 differentially expressed lncRNAs between GS and GU group.

LINC01224	0.237285	1.094898	2.206102	1.70E-05	0.000205
AC019171.1	0.268687	1.408641	2.390307	0.001037	0.005806
AP001107.9	2.225167	1.049407	-1.08434	2.78E-08	1.20E-06
UNC5B-AS1	0.62927	0.27905	-1.17315	2.62E-05	0.00029
AC124312.3	0.622759	0.287325	-1.11599	0.003071	0.013108
ATP13A4-AS1	1.614371	0.233035	-2.79235	2.95E-05	0.000321
AL355601.1	0.702778	0.276081	-1.34798	0.000146	0.001185
SCAT1	0.685115	1.520014	1.149666	1.52E-06	2.98E-05
AC012073.1	0.75505	1.793995	1.248532	1.10E-14	2.23E-11
ANKRD44-AS1	3.020619	0.910025	-1.73087	1.10E-08	6.56E-07
LINC02688	1.186479	0.301139	-1.97818	3.04E-06	5.26E-05
AC084864.1	0.329803	0.6737	1.030501	2.25E-06	4.14E-05
TBX2-AS1	1.168222	0.537863	-1.119	8.98E-09	5.51E-07
PRDM16-DT	0.942375	0.46155	-1.02981	1.51E-10	3.05E-08
LINC01942	0.829251	0.239431	-1.7922	3.89E-11	1.12E-08
AC099518.1	0.352194	0.97116	1.463337	2.99E-09	2.76E-07
AL512413.1	0.499429	1.082973	1.116645	0.012455	0.038191
AL136162.1	0.294144	0.674945	1.198246	5.96E-06	8.75E-05

DDN-AS1	0.330118	0.666443	1.013502	3.65E-08	1.48E-06
PTCSC3	4.052455	1.650436	-1.29595	5.86E-08	2.12E-06
AL109615.3	1.083154	5.017349	2.211687	1.82E-09	1.84E-07
SRGAP3-AS2	2.671768	0.882023	-1.59891	4.62E-06	7.15E-05
AL139023.1	0.089062	0.80752	3.180617	5.11E-06	7.61E-05
AL442224.1	0.32682	0.831228	1.346746	0.000277	0.002017
MIR193BHG	0.456571	1.034971	1.180678	1.99E-05	0.00023
AL162511.1	5.417999	2.265295	-1.25806	8.68E-09	5.50E-07
AL645608.1	0.244727	0.573426	1.228432	1.18E-05	0.00015
AC010789.1	0.227029	1.463641	2.688611	8.40E-09	5.50E-07
SFTA1P	43.93997	11.02874	-1.99427	1.02E-10	2.30E-08
ARHGEF2-AS1	0.926001	0.33296	-1.47567	8.70E-08	3.04E-06
MALINC1	1.376997	0.628312	-1.13197	0.001517	0.007922
AC073316.2	0.802686	0.362884	-1.14533	1.34E-06	2.65E-05
LINC02351	0.844695	0.321796	-1.39229	0.003696	0.015103
HHIP-AS1	3.765767	1.826197	-1.0441	3.83E-06	6.20E-05
AC013457.1	1.040204	0.360094	-1.53042	0.007373	0.02536
AC020907.1	0.585804	1.253359	1.097309	0.010275	0.032731
AC236972.3	0.863718	0.283698	-1.60621	2.76E-10	3.99E-08
ELFN1-AS1	0.232514	1.596231	2.779282	5.50E-08	2.03E-06
FAM83A-AS1	1.285841	3.697783	1.523948	3.25E-06	5.53E-05
AL445493.3	0.943164	0.234567	-2.00751	5.23E-09	4.24E-07
FENDRR	1.042873	0.316605	-1.71981	5.89E-07	1.40E-05
LINC00922	3.127204	0.807883	-1.95265	0.000107	0.000908
AL357093.2	1.850263	0.801334	-1.20725	0.000451	0.002988
AC099850.4	4.010727	8.875547	1.145972	5.98E-12	3.03E-09
AL021807.1	0.593839	1.551931	1.385921	0.000114	0.000957
LINC01644	1.445221	0.584124	-1.30694	4.35E-08	1.70E-06
AC089983.1	0.325706	1.5786	2.277004	4.03E-05	0.000416
AC120498.2	1.712576	0.642815	-1.41369	0.00996	0.03198
AP003119.3	0.411924	1.348213	1.710599	0.000107	0.000908
AC002070.1	0.982741	0.470945	-1.06125	1.93E-07	5.55E-06
LINC02446	1.267554	2.729716	1.106703	0.000299	0.002142
AP005262.1	0.143537	0.809695	2.495961	6.98E-06	9.82E-05
LINC02471	1.615229	0.707739	-1.19045	4.62E-05	0.000468
EGOT	0.847768	0.306458	-1.46798	6.06E-07	1.43E-05
AC110619.1	0.363331	1.024261	1.495227	0.002909	0.012701
LINC01504	1.082557	0.415546	-1.38136	5.10E-10	6.45E-08
AC005479.1	1.209447	0.552798	-1.12952	4.52E-07	1.12E-05
ST8SIA6-AS1	0.284897	1.253506	2.137459	0.00491	0.018877
AL079303.1	0.27991	0.748939	1.419886	0.000254	0.001892
AC016877.3	0.281356	0.900399	1.678169	6.87E-10	8.19E-08
AC110741.1	2.232929	0.168331	-3.72956	4.25E-07	1.08E-05

LINC02830	0.118859	0.79661	2.744625	0.000316	0.002232
LINC02038	2.948062	1.015257	-1.53792	5.32E-05	0.000513
LINC02163	0.168652	0.725614	2.105153	1.75E-06	3.39E-05
TRPM2-AS	0.349983	0.926726	1.404858	0.006961	0.024155
AP003119.2	0.609093	1.326604	1.123004	2.88E-05	0.000317
ERVE-1	0.659818	0.328676	-1.0054	1.90E-06	3.63E-05
LINC01765	0.894903	0.326694	-1.45379	8.91E-08	3.06E-06
AL138760.1	0.250477	0.906143	1.855063	0.01235	0.037969
LINC01270	0.763086	1.553506	1.02561	1.15E-08	6.66E-07
AC025154.2	2.907839	1.090018	-1.41559	3.17E-07	8.35E-06
BCAR4	0.011735	1.903049	7.34131	2.23E-08	1.10E-06
LINC01936	1.507704	0.62362	-1.27361	1.42E-07	4.37E-06
AC090772.3	0.43807	1.149939	1.392325	0.001984	0.009549
AC015722.2	0.798304	0.288614	-1.4678	0.000504	0.00329
LINC01088	1.302235	0.171989	-2.9206	0.000597	0.003802
LINC00337	0.216204	0.723234	1.74207	4.56E-11	1.15E-08
AC012213.4	0.184241	1.035916	2.491239	5.17E-06	7.65E-05
AC011944.1	1.183694	0.272135	-2.1209	2.45E-10	3.85E-08
LINC00342	17.23427	6.051934	-1.50981	4.72E-05	0.000471
AC079684.2	0.461044	0.935207	1.020382	1.16E-06	2.37E-05
AC112484.1	0.286285	0.596598	1.059308	0.003887	0.015689
AC015712.6	0.277981	0.59418	1.095914	0.016254	0.046911
AC084375.1	2.400583	0.929293	-1.36918	9.38E-08	3.17E-06
ELN-AS1	4.847552	2.271348	-1.09371	1.07E-09	1.21E-07
LINC01671	3.017523	1.30824	-1.20574	1.95E-07	5.55E-06
AP000251.1	0.351909	0.742243	1.076689	0.016765	0.047772
DIO3OS	0.796298	0.179193	-2.1518	4.21E-09	3.71E-07
LINC01214	0.343028	1.001741	1.546111	0.000268	0.001967
AC112722.1	0.60101	0.272668	-1.14024	4.93E-07	1.20E-05
LINC02864	0.354011	2.060582	2.541184	1.05E-07	3.48E-06
LINC02678	0.472097	0.962483	1.027678	0.001013	0.005716
C8orf34-AS1	5.959849	1.749108	-1.76866	1.27E-11	4.28E-09
LINC01133	3.620248	1.384668	-1.38655	0.000898	0.005201
MIR205HG	2.131974	0.695107	-1.61688	0.000113	0.000953
AL022313.2	0.315259	0.703362	1.157732	0.005889	0.021497
LINC01980	0.196833	1.476297	2.90694	5.08E-05	0.000495
AC005479.2	1.379669	0.656167	-1.07219	1.30E-07	4.11E-06
LINC01612	1.860756	0.24167	-2.94478	9.41E-12	3.81E-09
LINC00346	0.755045	1.662273	1.138522	6.90E-06	9.77E-05
AC010735.2	0.706289	1.461965	1.049578	0.000854	0.005002
LINC01535	0.260819	0.674629	1.371047	2.40E-06	4.35E-05
LHFPL3-AS2	4.633733	0.87474	-2.40525	2.08E-10	3.83E-08
LINC00524	0.824365	0.252768	-1.70547	4.92E-08	1.84E-06

AC087269.1	0.369588	0.804889	1.122873	0.003596	0.014747
RHOXF1-AS1	2.138922	0.779218	-1.45678	1.57E-08	8.39E-07
AC104031.1	2.944854	0.540945	-2.44464	1.51E-08	8.27E-07
AC080037.2	1.316915	3.093601	1.232124	8.86E-06	0.00012
MAFA-AS1	0.491869	1.100803	1.162211	0.002528	0.011407
MIR223HG	1.489147	0.695483	-1.0984	0.000469	0.003086
AP003119.1	0.318412	0.67485	1.083672	0.005869	0.02147
LINC02159	1.503864	0.630019	-1.25521	1.74E-07	5.17E-06
AC026369.3	1.494574	0.680402	-1.13528	0.001142	0.006304
LINC02195	0.912733	1.915816	1.069695	0.000387	0.002649
AC006206.2	0.324327	0.907707	1.484777	8.48E-06	0.000116
LINC00261	4.360525	1.623474	-1.42542	9.59E-13	9.71E-10
AC115099.1	0.648525	0.164014	-1.98334	0.001452	0.00772
AC013275.1	1.06534	0.491033	-1.11742	6.44E-06	9.28E-05
AC129926.1	0.213056	1.009638	2.244532	0.002096	0.009895
AC074135.1	0.484871	1.489975	1.619616	0.001171	0.006429
GATA6-AS1	0.893503	0.186993	-2.25649	1.17E-07	3.75E-06
AL024508.1	0.384433	0.836722	1.122016	7.43E-05	0.00068
LINC02588	0.237373	0.706191	1.572901	0.001861	0.009083
PARAL1	1.136966	0.51863	-1.13241	6.40E-06	9.28E-05
AC008268.1	10.23625	4.057053	-1.33518	4.84E-05	0.000478
LINC01833	0.291097	0.79654	1.452247	0.002075	0.009893
AC109454.3	0.051748	0.766676	3.889041	0.003427	0.014257
LINC00665	2.055403	4.796751	1.222636	2.47E-10	3.85E-08
BX640514.2	1.456023	0.708446	-1.0393	7.10E-09	5.23E-07
AL031058.1	0.918552	2.62792	1.516488	3.01E-12	2.03E-09
AC000061.1	1.200516	0.076289	-3.97603	0.000677	0.004207
UBXN10-AS1	3.085866	1.338249	-1.20533	2.48E-08	1.14E-06
LINC00942	0.299537	6.173492	4.365283	0.000212	0.001643
DSCR8	0.139314	1.123405	3.01147	1.46E-05	0.000178
LINC01518	0.213362	0.935212	2.13199	5.90E-05	0.000564
LINC02122	0.723744	0.238778	-1.59981	3.17E-06	5.45E-05
KCNMB2-AS1	0.775776	1.881364	1.278068	0.001975	0.009529
LINC02555	1.72079	0.336739	-2.35337	3.92E-05	0.000411
AF186192.3	0.138855	0.758481	2.449532	0.008868	0.029032
AL590226.1	0.767719	0.301174	-1.34998	1.29E-06	2.58E-05
LINC02772	0.92717	0.210733	-2.13742	8.90E-07	1.94E-05
AC061975.6	0.118732	0.792799	2.739247	3.71E-06	6.08E-05
AC010998.3	1.001269	0.254372	-1.97682	4.00E-05	0.000416
AC011447.3	0.288697	0.755365	1.38762	0.0021	0.009895
AL031667.3	0.335765	0.741401	1.142802	0.000157	0.001264
AP005233.2	1.105763	2.595373	1.230901	0.002099	0.009895

GS, genomic stable; GU, genomic unstable.

Supplementary Table S2. The significantly enriched pathways in the high-risk group of early-stage LUAD patients.

NAME	SIZE	NES	NOM p-value	FDR q-value
KEGG_PROTEASOME	46	2.300	0.000	0.0015
KEGG_CELL_CYCLE	124	2.246	0.000	0.0025
KEGG_PYRIMIDINE_METABOLISM	97	2.185	0.000	0.0034
KEGG_ALZHEIMERS_DISEASE	165	2.131	0.002	0.0054
KEGG_PATHOGENIC_ESCHERICHIA_COLI_INFECTION	56	2.014	0.000	0.0130
KEGG_PENTOSE_PHOSPHATE_PATHWAY	27	2.002	0.000	0.0136
KEGG_HOMOLOGOUS_RECOMBINATION	28	2.026	0.000	0.0142
KEGG_OOCYTE_MEIOSIS	112	2.034	0.000	0.0147
KEGG_DNA_REPLICATION	36	1.972	0.000	0.0147
KEGG_SPLICEOSOME	127	1.949	0.010	0.0159
KEGG_MISMATCH_REPAIR	23	1.958	0.002	0.0161
KEGG_HUNTINGTONS_DISEASE	180	1.973	0.010	0.0162
KEGG_CITRATE_CYCLE_TCA_CYCLE	31	1.913	0.000	0.0206
KEGG_PURINE_METABOLISM	158	1.894	0.000	0.0234
KEGG_UBIQUITIN_MEDIATED_PROTEOLYSIS	134	1.881	0.004	0.0254
KEGG_NUCLEOTIDE_EXCISION_REPAIR	44	1.850	0.006	0.0314
KEGG_PARKINSONS_DISEASE	128	1.842	0.022	0.0315
KEGG_RNA_DEGRADATION	59	1.835	0.004	0.0317
KEGG_OXIDATIVE_PHOSPHORYLATION	131	1.811	0.033	0.0343
KEGG_RIBOFLAVIN_METABOLISM	16	1.803	0.006	0.0352
KEGG_AMINOACYL_TRNA_BIOSYNTHESIS	41	1.811	0.015	0.0359
KEGG_PYRUVATE_METABOLISM	40	1.814	0.002	0.0368
KEGG_GLYCOLYSIS_GLUCONEOGENESIS	62	1.781	0.004	0.0385
KEGG_PROTEIN_EXPORT	24	1.770	0.010	0.0390
KEGG_RNA_POLYMERASE	28	1.775	0.010	0.0390
KEGG_CYSTEINE_AND_METHIONINE_METABOLISM	34	1.765	0.004	0.0391
KEGG_N_GLYCAN_BIOSYNTHESIS	46	1.784	0.016	0.0391
KEGG_GLYOXYLATE_AND_DICARBOXYLATE_METABOLISM	16	1.730	0.024	0.0497

LUAD, *lung adenocarcinoma; NES, normalized enrichment score; NOM, nominal; FDR, false discovery rate.*
